# Effect of an adjustable ceiling to prevent premature rising attempts after general anesthesia in healthy ponies and horses: A pilot study

**DOI:** 10.1111/vsu.14181

**Published:** 2025-01-30

**Authors:** Anna Lindqvist, Görel Nyman, Anneli Rydén, Ove Wattle

**Affiliations:** ^1^ Swedish University of Agriculture Science Uppsala Sweden

## Abstract

**Objective:**

This study aimed to assess and compare the recovery of ponies and horses following general anesthesia in two different settings: a recovery box with an inflexible, adjustable ceiling, and free recovery without restraints. Our primary objective was to evaluate the effect of adjustable ceilings on the prevention of premature attempts to rise during recovery. The secondary aim was to compare the physiological stress indicators during recovery.

**Study design:**

Prospective, randomized, experimental study.

**Animals:**

Six healthy ponies and 10 healthy horses.

**Methods:**

This study used a crossover design with two settings: an inflexible, adjustable ceiling and free recovery. Recovery was scored using a quality scoring system. Heart rate, and lactate, glucose, and cortisol levels were analyzed and compared between the animals and recoveries.

**Results:**

All animals had a higher recovery quality (*p* = .026) with an adjustable ceiling than with free recovery. No differences were observed in glucose level or heart rate between the two settings. However, horses had higher blood lactate, 2.9 ± 1.2 mmol/L versus 1.6 ± 0.7 mmol/L (*p* = .025), and serum cortisol, 184 ± 81 nmol/L versus 93 ± 20 nmol/L (*p* = .031) in restricted recovery than free recovery, respectively.

**Conclusion:**

An inflexible, adjustable ceiling improved the quality of recovery and prevented premature rising attempts; however, it was associated with increased lactate and cortisol levels, indicating an increased level of stress.

**Clinical significance:**

Limiting premature rising attempts with an adjustable ceiling during recovery phase has the potential to improve the quality of recovery in horses. Further research is needed to draw conclusions for clinical use.

**Abbreviations**
IM: intra muscular
IV: intra venously
kPa: kilo pascal

## 1 INTRODUCTION

The recovery phase is critical for horses under general anesthesia as serious complications can occur, such as fractures, joint dislocations, respiratory obstruction, neuropathies, or myopathies.^1^ The mortality rates have been reported to be 0.6% to 1.6% and 0.9% to 3.4% in non‐colic‐related and colic‐related surgeries, respectively, of which 25.6% were caused by recovery‐associated fractures.^2^, ^3^, ^4^ The reported mortality rate in horses is high in comparison with dogs (0.17%), cats (0.24%),^5^ and humans (0.001%).^6^

Several methods for assisted equine recovery have been developed to reduce the risk of injury, such as headtail rope,^7^, ^8^ sling,^9^ and hydropool systems,^10^, ^11^ tilt tables,^12^ and inflated air pillows.^13^ All manual handling of a horse during its recovery might involve staff safety issues.^4^ The head‐and‐tail rope method, the most common form of assisted recovery, has advantages and disadvantages.^7^, ^8^, ^14^, ^15^ The method is designed to assist when the horse rises spontaneously; however, it cannot prevent premature and possibly harmful rising attempts. Methods such as hydropool systems, tilt tables, inflatable pillows, and Anderson slings have shown some advantages; however, all require trained personnel (two or more) and proper facilities.^10^, ^11^, ^12^, ^13^

This study aimed to assess and compare the recovery of ponies and horses following general anesthesia in two different settings: a recovery box with an inflexible, adjustable ceiling and free recovery without restraints. Earlier pilot studies reported that ponies and horses accept forced recumbency as a possible adjunctive treatment to unload the hooves in the acute phase of equine laminitis.^16^, ^17^ To handle unforeseen events involving both the animals and roof prototype, it was suggested, for safety reasons, to start by handling ponies rather than large horses. Our primary objective was to evaluate the effect of an adjustable ceiling on the prevention of premature attempts to rise during recovery. The secondary aim was to compare the physiological stress indicators during recovery.

We hypothesized that recovery with an adjustable ceiling would prevent premature rising attempts in the early recovery period, thereby improving the quality of recovery without increasing objective measures of stress compared to free recovery.

## 2 MATERIALS AND METHODS

The study was conducted with ponies and horses in two different settings: free recovery without restraints, and a recovery box with an inflexible, adjustable ceiling.

### 2.1 Animals

This study included six healthy Shetland ponies and 10 horses (eight warm‐blooded trotters and two Swedish Warmbloods) (Table 1). All animals were obtained from the university's teaching herd and the study was approved by the ethical committee for animal experiments in Uppsala, Sweden (No. 5.8 18‐05, 181/2019). For ponies, the mean age was 13.3 years, with a range of 10–17 years, and the mean weight was 180 kg, with a range of 149 to 275 kg. For horses, the mean age was 11 years with a range of 8–20 years, and the mean weight was 539 kg, with a range of 457–640 kg. The thoracic heights of the animals (from the sternum to the withers) were measured before anesthesia to determine the intended height of the ceiling. The measured heights were 46–58 cm for ponies and 75–85 cm for horses. All animals were housed in the department's stables and outdoors in a paddock during daytime. The animals were made to fast for 12 h before anesthesia; however, they were allowed water *ad libitum*.

**TABLE 1:** Recovery quality score 0–5 devised by Young and Taylor.^18^

Score 5	No ataxia, no struggling, stood up at first attempt as if fully conscious
Score 4	Slight ataxia and staggering, stood at first or second attempt, no serious instability
Score 3	Some staggering and ataxia, a few unsuccessful attempts to stand, ataxic immediately after standing up
Score 2	Excitement, paddling when recumbent, several attempts to stand, severe ataxia once standing, may fall, danger of self‐inflicted injury
Score 1	Excitement when recumbent, persistent unsuccessful attempts to stand, severe ataxia and falls once standing, aimless waking, high risk of self‐inflicted injury
Score 0	Very violent, self‐inflicted injury, prolonged struggling or unable to stand 2 h after the end of anesthesia

Six horses were included in a parallel randomized study investigating subcutaneous reactions to absorbable sutures used for small skin incisions (≤ 4 cm) on their trunks, in line with the animal ethics aim of reducing horse numbers in clinical studies.

### 2.2 Anesthesia

All animals were examined, and an American Society of Anesthesiologists (ASA) physical status, I–V, was determined. A minimum of 2 weeks passed between anesthesia administrations to allow the metabolites to wash out after each anesthesia.^19^ The animals were premedicated with acepromazine (0.03 mg/kg IM) and meloxicam (0.6 mg/kg IV). A 14 gauge catheter was placed aseptically in one of the jugular veins after applying a topical local anesthesia containing lidocaine and prilocaine (Emla, Aspen Pharma) (25 mg/g + 25 mg/g), and a subcutaneous mepivacaine injection (2 mL) (20 mg/mL). The animals were sedated with romifidine (0.1 mg/kg IV) and butorphanol (0.03 mg/kg IV), and sponge earplugs were fitted during experiments. General anesthesia was induced using midazolam (0.05 mg/kg IV) and ketamine (2.2 mg/kg IV). Inadequate anesthetic depth occurred in one pony and three horses; therefore, thiopental (0.5 mg/kg IV) was also administered. Once the animals were unconscious, the trachea was intubated and they were placed in right lateral recumbency on a padded surgery table with their right front limb pulled forward. Leg support was provided to ensure optimal positioning of the patient on the surgical table. A sterile urine catheter was placed to empty the urinary bladder.

Anesthesia was maintained using isoflurane (EtISO 1.3% to 1.7%) in oxygen (50%) and air (50%) delivered via a large animal anesthetic circuit (Tafonius large animal ventilator, Vetronic Services Ltd, Devon, UK.). Volume‐controlled mechanical ventilation was used to maintain partial pressure of carbon dioxide from below 5 kilo pascal (kPa) to 7 kPa. A catheter was placed in a branch of the transverse facial artery or in the facial artery for invasive blood pressure monitoring. During anesthesia, Ringer's solution was administered intravenously at a maintenance rate (2 mL/kg/h). Hypotension (mean arterial pressure < 70 mmHg) occurred in six horses and was treated with a continuous intravenous infusion of dobutamine (0.5 μg/kg/min IV) until resolved. The large‐animal anesthesia delivery system monitored and recorded all relevant data during anesthesia, such as the animals' temperature, oxygen saturation, heart rate, and respiratory rate, as well as showing a capnogram, electrocardiogram, and blood pressure.

Anesthesia was maintained for 120 min in both ponies and horses. Xylazine (0.2 mg/kg IV) was administered when the animals were disconnected from the anesthesia circuit.^20^ The animals were moved to the recovery box using a hoist and placed in right lateral recumbency. A nasopharyngeal tube was placed in one nostril and oxygen was delivered through the tube at 10–12 L/min until the animals moved their heads. The patients were extubated after the return of the swallowing reflex.

### 2.3 Recovery

The ponies and horses were allowed to recover in two different settings: free recovery and a box with an inflexible adjustable ceiling. The order of recovery was randomized by flipping a coin. The ceiling for the ponies was lowered for 30 min. The time was determined based on a previous anesthesia study at the university, in which most ponies had recovered within half an hour.^21^ The inflexible ceiling was lowered for 70 min for the horses. The ceiling was elevated by one person. The time was determined based on a previous study reporting recovery time to standing in horses as 54–77 min.^22^ Each recovery was monitored, and the number of rising attempts was noted manually. The animals' heart rates were recorded continuously using a Polar M430 heart rate monitor and a Polar H10 chest strap sensor (Polar Electro Oy, Kempele, Finland).

**FIGURE 1:**
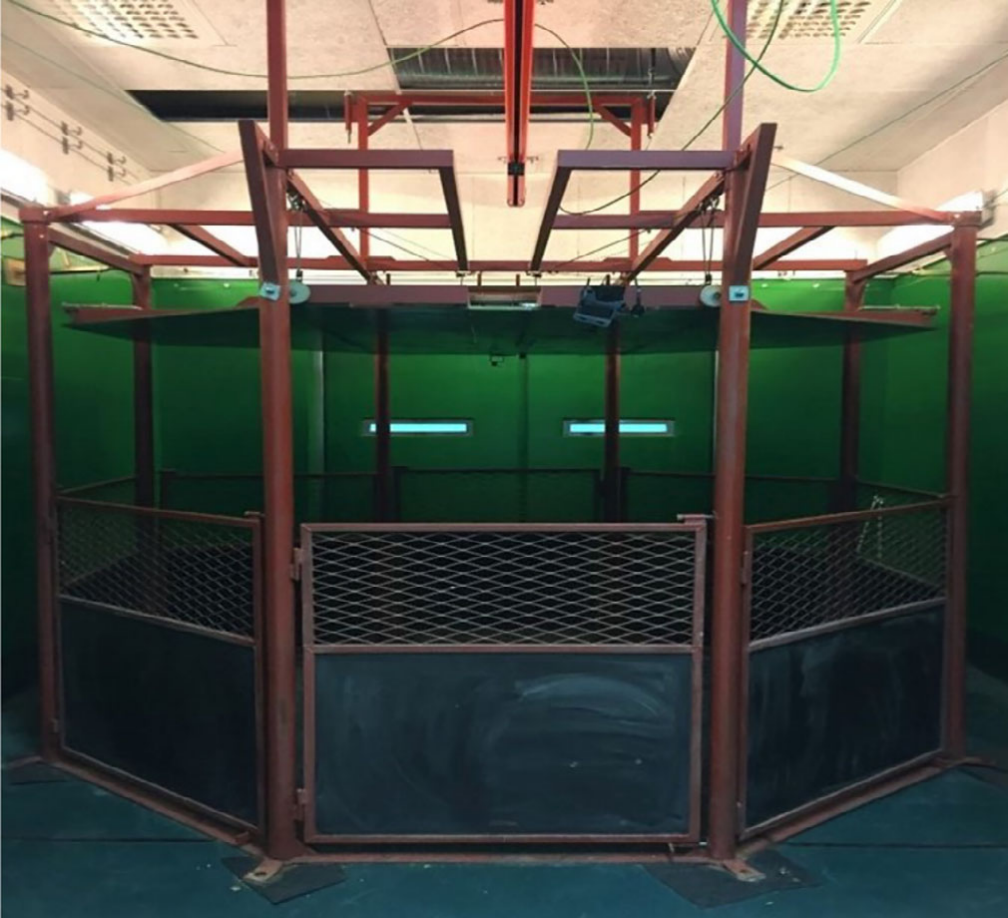
The box with an inflexible, adjustable ceiling.

Free recovery occurred in one of the two padded rectangular recovery boxes with a nonslip floor and rounded corners, measuring 14.6 m^2^ in area. The inflexible ceiling recovery box (Figure 1) was a regular octagon‐shaped recovery box with an area of 8.3 m^2^. Each of the eight Masonite walls had an independent door to allow access in all directions. An electric winch was used to adjust the ceiling height. An inflexible ceiling was installed in the recovery room equivalent to that used for free recovery. The box was constructed by the last author and manufactured at the Swedish University of Agriculture Science. The height of the ceiling was set initially at 1.15 times the pony's sternal height. The ceiling height was adjusted to allow the animals to lie in a sternal position with their heads and necks in a natural position. The height was set at 1.15 times the sternal height for the first horse and at the sternal height for the remaining nine horses. A rising attempt was defined as when the animal's thorax was lifted from the floor for free recovery and when the animal tried to lift the ceiling during restricted recovery.

### 2.4 Blood sampling

Blood samples were collected and analyzed in a similar manner for all animals and recovery settings. Blood was sampled from the catheter into three different tubes (serum, ethylenediaminetetraacetic acid, and heparin) before induction, 30 min after induction, after anesthesia, and when the animals stood up safely after recovery. The glucose and lactate levels were measured immediately after sampling using calibrated glucose (Accu‐Check Aviva; Roche Diagnostics Scandinavia AB, Solna, Sweden) and lactate (Lactate Pro2 LT‐1730; Arcray, Amstelveen, Netherlands) devices. The remaining blood samples were centrifuged and frozen to −80°C in Eppendorf tubes. Serum cortisol levels were analyzed in duplicate using Immulite 2000XPi (Siemens Healthcare Diagnostics, Munich, Germany).

### 2.5 Recovery scoring

All animals were scored by the first author using Young and Taylor's 0–5 scoring system (Table 1).^18^

### 2.6 Statistics

Parametric data are presented as means and standard deviations, whereas nonparametric data are presented as ranges and median values. The normality of all data was assessed using the Shapiro–Wilk test. Statistical significance was set at *p* < .05. All statistical analyses were performed using RStudio Version 0.99.903 (RStudio Inc., Boston, Massachusetts). This study was exploratory in nature, and, due to the lack of previously published data, no statistical power calculations could be performed to determine the sample size.

Differences in recovery scores were analyzed using the Wilcoxon test. A paired two‐sample *t*‐test was used to determine the differences in the animals' glucose, lactate, and cortisol levels, and heart rates. The Wilcoxon test was used to determine the differences in the lactate, glucose, and cortisol levels, heart rate, and recovery scores between the six horses that participated in the absorbable suture study and the four horses that did not.

A mixed model analysis of variance (ANOVA) was used to determine the differences in the animals' heart rates during the four physical positions/activities: lateral position, sternal position, rising attempts, and standing.

## 3 RESULTS

All animals completed the study and received an ASA status 1 score.^23^ No adverse post anesthesia effects were observed.

### 3.1 Recovery from anesthesia

Table 2 shows the individual data. In free recovery, the time to a standing position was 37 ± 18 min for ponies and 46 ± 13 min for horses. The median number of rising attempts, including successful attempts, was 2.5 (range: 1–5) for ponies and 1.7 (range: 1–3) for horses. Three ponies and four horses stood up on their first rising attempt 36–58 min after extubation. Three ponies and one horse scored 2 out of 5 points, as they made several attempts to stand and had severe ataxia upon standing, with a risk of falling or self‐inflicted injury. These animals made their first attempt to rise 13–40 min after extubation, with two to five unsuccessful rising attempts before standing.

**TABLE 2:** Time (min) to extubation, first rising attempt, number of rising attempts, and time to standing.

	Free recovery					Inflexible ceiling				
	Time to extubation	Time to first rising attempt	Rising attempt	Time to standing	RS	Time to extubation	Time to first rising attempt	Rising attempt	Time to standing	RS
H1♀	11	20	2	30	4	9	14	3	42^a^	4^b^
H2♀	9	40	1	40	4	7	13	5	70	5
H3♀	7	40	2	57	2	6	12	10	70	5^b^
H4♂	10	24	2	53	3	8	28	9	71	4^b^
H5♂	9	38	1	38	4	5	26	7	70	5
H6♀	8	32	2	52	3	5	59	6	76	5
H7♀	7	10	3	18	3	6	45	5	70	4
H8♀	9	47	2	58	4	5	35	5	72	5
H9♀	8	54	1	54	3	7	48	5	70	5
H10♀	9	58	1	58	4	7	28	7	70	4
P1♂	6	13	5	17	2	6	15	2	30	4^b^
P2♀	2	12	4	14	2	4	12	4	30	4
P3♂	6	59	1	59	4	4	57	1	57	5
P4♂	2	58	1	58	3	3	56	1	56	4
P5♂	10	17	4	36	2	6	28	2	30	3
P6♀	8	36	1	36	4	6	36	1	36	4

*Note*: Time to extubation after entering the recovery box, time to standing after extubation, and time to standing are given in minutes. The number of rising attempts included the final successful attempts. Recovery scores: A higher score (maximum of 5) indicated better recovery quality. H1–H6 (inflexible ceiling recovery) also participated in the absorbable suture study.

Abbreviations: ♂, gelding; H, horse; ♀, mare; P, pony; RS, recovery score.

^a^ Ceiling elevated before the restriction time due to technical difficulties.

^b^ Additional thiopental was administered in induction.

During recovery with an inflexible ceiling, all animals were restricted by the inflexible ceiling despite the attempts to rise. The time from elevating the ceiling to the standing position was 0–27 min for ponies and 0–6 min for horses. The inflexible ceiling had to be elevated prematurely after 42 min for one horse due to technical difficulties with the ceiling, and the horse stood up immediately on the first attempt. Three of six ponies and six of 10 horses stood immediately after the ceiling was elevated, whereas the remaining animals were recumbent for 1–26 min after the ceiling was raised. The median number of rising attempts, including nonrestricted successful attempts, was 1.5 (range: 1–4) for ponies and 5.5 (range: 3–10) for horses. All animals had recovery scores of 4 or 5 when rising, with a median of 4 (range: 3–5) for ponies and 4.6 (range 4–5) for horses. All animals with an inflexible ceiling had better recovery scores than those with free recovery (*p* = .026). Three horses and one pony received additional thiopental because of inadequate anesthetic depth after induction of anesthesia. These animals did not show any deviation in the time to the first rising attempt or recovery scores.

### 3.2 Physiological stress indicators

The mean lactate levels after recovery with the inflexible ceiling (2.1 ± 0.4 mmol/L) were not higher than those in free recovery (2.4 ± 0.7 mmol/L) (*p* = .6) for the ponies. The mean glucose levels were 7.4 ± 1.8 mmol/L with the inflexible ceiling, and 8.7 ± 1.9 mmol/L with free recovery (*p* = .12). The cortisol level after free recovery was 78 ± 61 nmol/L (Table 3). However, due to technical difficulties with the samples from the ponies in the inflexible ceiling setting, the cortisol values could not be analyzed.

**TABLE 3:** Physiological stress indicators.

	Lactate mmol/L		Glucose mmol/L		Serum cortisol nmol/L^a^		Heart rate mean BPM	
	Horses	Ponies	Horses	Ponies	Horses	Ponies	Horses	Ponies
Free recovery	1.6 ± 0.7	2.4 ± 0.4	10.9 ± 1.6	8.50 ± 1.6	93 ± 20^b^	69 ± 25	44 ± 6	33 ± 8
Inflexible ceiling	2.9 ± 1.2	2.1 ± 0.4	10.7 ± 3.1	7.4 ± 1.8	184 ± 81^c^	N.A.	44 ± 3	37 ± 8

*Note*: Mean and standard deviation of lactate, glucose, and cortisol levels while standing after recovery, and heart rate during the entire recovery period in ponies (*n* = 6) and horses (*n* = 10) in the free recovery setting and setting using an adjustable, inflexible ceiling.

Abbreviations: BPM, beats per minute; N.A., not analyzed.

^a^ University hospital clinical pathology laboratory. Reference value for healthy horses: below 320 nmol/L.

^b^ Serum cortisol analyzed in five horses.

^c^ Serum cortisol analyzed in six horses.

In horses, higher lactate and cortisol levels were found between recovery settings. The mean lactate value was 2.9 ± 1.2 mmol/L after recovery with the inflexible ceiling, compared with free recovery level of 1.6 ± 0.7 mmol/L (*p* = .025). The mean cortisol level with an inflexible ceiling was 184 ± 81 nmol/L, compared with 93 ± 20 nmol/L for free recovery (*p* = .031). The glucose levels did not differ between free recovery (10.9 ± 1.6 mmol/L) and recovery with an inflexible ceiling (10.7 ± 3.1 mmol/L) (*p* = .806).

The mean heart rate during the entire recovery period did not differ between ponies (*p* = .773) and horses (*p* = .867). The mean heart rate (beats per minute, BPM) for all animals during recovery with an inflexible ceiling did not differ between lateral recumbency (37 BPM), sternal recumbency (40 BPM), restricted rising attempts (43 BPM), and standing (48 BPM) (*p* = .996).

There were no differences in lactate (*p* = .45), glucose (*p* = .07), and cortisol (*p* = .27) levels, heart rate (*p* = .39), or recovery score (*p* = .7) between the six horses that participated in the absorbable study and the four horses that did not.

## 4 DISCUSSION

One reason for this study was to evaluate whether an adjustable ceiling prevented nonphysiological loading of the limbs and ataxia when equids stood up during recovery after general anesthesia. The 0–5 scoring system was used because it is considered to provide a good and rapid overview and is easily applied.^24^ The scoring system is also the one used for all recoveries at our equine hospital, at the Swedish University of Agriculture Science. All animals that were restricted from making premature‐rising attempts with an adjustable ceiling had better recovery scores than those with free recoveries. The scores of all horses and five of the six ponies indicated a good recovery with the inflexible ceiling in comparison with free recoveries, where five horses and four ponies scored unsatisfactory to poor recoveries.^18^

Fracture surgery has a high risk of complications during recovery, and methods to ensure safe recovery without unphysiological loading of the treated limb are required.^3^ This study was started with the inflexible ceiling height set at 1.15 times the sternal height for the first horse, as this has been reported as acceptable for horses under forced recumbency when used as an adjunct in the treatment of acute equine laminitis.^16^, ^17^ In the same horse, it was observed that the height of the inflexible ceiling needed to be adjusted to 1.0 times the height of the chest to prevent the horse from placing a load on its limbs during a premature rising attempt. For this horse, the ceiling was elevated prior to the determined 70 min because of technical difficulties (which were resolved for the remaining horses).

Another objective of this study was to compare the physiological indicators of stress during different recovery periods (Table 3). Heart rates have commonly been used as potential parameters to estimate stress during transportation.^25^, ^26^ Heart rates were not higher in any of the four compared events: lateral recumbency, sternal recumbency, attempts to rise or stand, and recovery using an inflexible ceiling. The animals had lower heart rates during the rising attempts than during the standing attempts, suggesting that recovery itself created stress.

Lactate levels have been reported to be elevated after general anesthesia, and many factors can affect the results, such as drugs and position during anesthesia.^19^, ^27^ Regardless of how many rising attempts the horses in this study made, the lactate levels after standing were higher with the inflexible ceiling than those in the free‐recovery setting. Owing to the limited number of horses used, it was difficult to determine whether this lactate elevation was a result of the horses' rising attempts, lying down for a longer period, or only a normal variation after 2 h of anesthesia. The mean lactate level when standing in the inflexible ceiling setting was 2.9 ± 1.2 mmol/L, a level that is higher in healthy horses compared with previously presented results.^28^ The mean lactate level at rest has been measured to be 2.3 ± 1.2 mmol/L in show jumpers at a competition facility.^29^ The recovery time (70 min) and more rising attempts (Table 2) in the horses under the inflexible ceiling may have reduced the muscle perfusion, increased the efforts, and therefore elevated the lactate values. Increased lactate values were not observed in ponies in which the time limit under the ceiling was 30 min, and they also had fewer rising attempts.

Increased glucose levels can be seen both after general anesthesia and as a result of sedation with alpha‐2‐agonists; however, they have also been used as a marker for stress after transportation and competition.^30^, ^31^, ^32^ The reported levels have been measured to be 5.4 mmol/L after transportation and 12 mmol/L after an alpha‐2 agonist was administered.^30^, ^33^ The increased glucose levels in this study were probably influenced by sedation with alpha‐2 agonists; therefore, glucose as a marker for stress is difficult to interpret. Further studies on compromised horses are required to draw definitive conclusions.

Higher postrecovery mean cortisol levels were found in horses in the inflexible ceiling recoveries (184 ± 81 nmol/L) compared with free recoveries (93 ± 20 nmol/L), indicating that the inflexible ceiling or the time spent under a ceiling (70 min) caused more stress. According to the clinical pathology laboratory in the university hospital, the reference value for cortisol in healthy horses is below 320 nmol/L. All cortisol levels in this study were therefore within the reference limits. Contradictory conclusions exist regarding the use of cortisol levels as a marker of stress in clinical settings. Cortisol levels have been found to be elevated after general anesthesia (> 350 nmol/L) and in equine competition.^19^, ^34^, ^35^ Cortisol levels have also been found to be elevated in horses during transportation (up to 325 nmol/L),^36^ an event involving restriction of the horses' natural behavior that may transmit to the restriction in recovery with an inflexible ceiling.^25^, ^36^, ^37^, ^38^ For horses with compromised welfare, cortisol is a poor marker for stress.^34^, ^39^ In summary, cortisol level is only one indicator of stress in horses and may be a suboptimal marker for stress. Thus, it is difficult to draw substantive conclusions from our data.

In humans, the elimination half‐life of isoflurane in the second phase, for example elimination from well perfused organs such as the brain, is 19.4 ± 7.7 min.^40^ As far as the authors are aware, no studies have been published in which the optimal recovery time after general anesthesia or the elimination half‐life of isoflurane have been determined in horses. If the same emilination time is applied to horses, the elimination time would be 35–40 min. One study reported that ponies had a mean time of 36 min to stand, indicating that there are no obvious differences between ponies and horses.^41^ Sedation with xylazine (0.6 mg/kg) has been shown to have a half‐life of 45–50 min.^42^ In healthy ponies and horses, the restriction time in this study (30 or 70 min) might be too short or long. Three horses and one pony received additional thiopental when inadequate anesthetic depth occurred after the induction of anesthesia (Table 2). These animals did not show any deviation in the time to the first rising attempt or recovery scores. It is important to note that additional thiopental administration during anesthesia may influence recovery.^43^ However, as the length of inhalation anesthesia was 120 min, the additional thiopental administered had no impact on the time to standing or recovery quality.

Six horses participated in the absorbable suture material study. The “Replacement, Reduction and Refinement” principle for clinical trials involving animals was used to minimize pain and suffering. There were no differences in the stress markers or recovery scores between these horses and the four horses that did not participate. It has been suggested that mild surgical procedures (“minor body surface surgery”) have no effect on the quality of recovery, whereas fracture repair, exploratory laparotomies, and ocular surgery have an increased risk of poorer recovery after general anesthesia.^44^ However these six horses recovered with an adjustable ceiling; therefore, no clear and definitive conclusion can be made regarding its impact.

This study had some limitations. Blind scoring was impossible because the recovery setting was obvious when the recoveries were reviewed and evaluated from the recordings. Other scoring systems could be used for comparison in future studies.^24^ This study included only 16 healthy animals, making it difficult to draw any conclusions regarding recovery differences between breeds and types of surgical interventions. To achieve an 80% power in the difference for heart rate and blood glucose, more than 400 horses would be required. The cortisol values in all horses and ponies in all recovery settings could not be analyzed, which would provide more data to further evaluate the stress levels. To draw more precise conclusions, all the 16 animals should have been subjected to superficial wounds. An additional limitation was that the animals should have received a redose of ketamine instead of thiopental during an event of inadequate anesthetic depth, to standardize the anesthetic protocol.

Implementing a recovery approach for horses and ponies, including an adaptable ceiling, could potentially enhance post‐general‐anesthesia safety and prevent premature attempts to rise. In contrast to free recovery, a recovery ceiling for healthy animals demonstrated an improved quality of recovery without marked variations in heart rate or glucose levels. Elevated cortisol and lactate levels suggest that an inflexible ceiling during recovery could induce more stress than a free recovery. One advantage of using an adjustable ceiling to improve the quality of recovery is that only one person is required to raise the ceiling when it is time to allow the horse to stand. The box also enabled access to the horse from eight different directions if any complications occurred or if the horse required assistance. Thus, a ceiling can be considered as a good complement to the toolbox for improved recovery. However, further investigation into the postoperative recovery of horses is essential before formulating any conclusive recommendations.

## CONFLICT OF INTEREST

The authors declare no conflicts of interest related to this report.
